# Enhancing the yield of Xenocoumacin 1 in *Xenorhabdus nematophila* YL001 by optimizing the fermentation process

**DOI:** 10.1038/s41598-024-63794-2

**Published:** 2024-06-12

**Authors:** Yunfei Han, Shujing Zhang, Yang Wang, Jiangtao Gao, Jinhua Han, Zhiqiang Yan, Yongquan Ta, Yonghong Wang

**Affiliations:** 1 Key Laboratory of Plant Protection Resources and Pest Management, Ministry of Education, College of Plant Protection, Northwest A&F University, 22 Xinong Road, Yangling, 712100 Shaanxi China; 2 Key Laboratory of Green Prevention and Control of Tropical Plant Diseases and Pests, Ministry of Education, School of Tropical Agriculture and Forestry (School of Agricultural and Rural Affairs, School of Rural Revitalization), Hainan University, 58 People’s Avenue, Haikou, 570228 Hainan China; 3 Shaanxi Research Center of Biopesticide Engineering & Technology, College of Plant Protection, Northwest A&F University, 22 Xinong Road, Yangling, 712100 Shaanxi China

**Keywords:** Xenocoumacin 1 (Xcn1), Fermentation optimization, *Xenorhabdus nematophila*, Biopesticide, Response surface methodology, Biotechnology, Microbiology

## Abstract

Xenocoumacin 1 (Xcn 1), antibiotic discovered from secondary metabolites of *Xenorhabdus nematophila*, had the potential to develop into a new pesticide due to its excellent activity against bacteria, oomycetes and fungi. However, the current low yield of Xcn1 limits its development and utilization. To improve the yield of Xcn1, response surface methodology was used to determine the optimal composition of fermentation medium and one factor at a time approach was utilized to optimize the fermentation process. The optimal medium composed of in g/L: proteose peptone 20.8; maltose 12.74; K_2_HPO_4_ 3.77. The optimal fermentation conditions were that 25 °C, initial pH 7.0, inoculum size 10%, culture medium 75 mL in a 250 mL shake flask with an agitation rate of 150 rpm for 48 h. *Xenorhabdus nematophila* YL001 was produced the highest Xcn1 yield (173.99 mg/L) when arginine was added to the broth with 3 mmol/L at the 12th h. Compared with Tryptic Soy Broth medium, the optimized fermentation process resulted in a 243.38% increase in Xcn1 production. The obtained results confirmed that optimizing fermentation technology led to an increase in Xcn1 yield. This work would be helpful for efficient Xcn1 production and lay a foundation for its industrial production.

## Introduction

*Xenorhabdus nematophila* is an entomopathogenic nematode symbiotic bacteria which belonging to the family *Morganellaceae*^1,2^ Generally, *X. nematophila* has two phenotypic variants, called primary and secondary. Only the primary variant absorbed bromothymol blue in the nutrient agar media supplemented with 25 mg/L bromothymol blue and 40 mg/L triphenyltetrazolium chloride (NBTA)^2,3^. Significantly, the primary variant produced various antimicrobial secondary metabolites such as nematophin^4^, odilorhabdins^5^, pristinamycin^6^, xenocoumacins^7^, and xenortides^8^. Xenocoumacin 1 (Xcn 1), a water-soluble antimicrobial agent produced by *Xenorhabdus nematophila*, originally identified in the fermentation broth of *Xenorhabdus* sp. Q1 (ATCC 39497)^7^. Biosynthetic gene cluster of Xcn1 had been identified that the production of Xcn1 was generated by *xcnA-L* genes and disintegrated by *xcnMN* genes^9,10^.

Pharmacological studies on Xcn1 showed that it had antibacterial and antifungal activity. The minimum inhibitory concentration (MIC) were 0.5 μg/mL and 0.125 μg/mL when Xcn1 against *Escherichia coli* ESS and *Cryptococcus neoformans*, respectively^7^. Xcn1 also showed biological activity with median effect concentrations (EC_50_) ranging from 0.25 to 4.17 μg/mL against five species of *Phytophthora*^11^. Furthermore, Xcn1 had the potential to be developed as an acaricide. The half maximal inhibitory concentration (IC_50_) of Xcn1 against *Tetranychus urticae* was 17.71 μg/mL^12^. Interestingly, Xcn1 was friendly to *Neoseiulus californicus*, the natural enemy of *T. urticae*^13^. Therefore, the development of Xcn1 as a new pesticide was a possibility.

However, the low yield and high cost of chemical synthesis of Xcn1 limit its industrial production. Approximately 7–9 steps were required to chemically synthesize Xcn1^14^. Similarly, the low yield of Xcn1 produced by *X. nematophila* was a serious limitation to its commercialization. Therefore, it was necessary to improve the production of Xcn1. Several strategies, aiming at improving the biosynthetic efficiency, such as tuning the expression of regulatory genes, increasing the supply of precursors, and over-expressing the biosynthesis and export genes, were used to increase antibiotic yields^15,16^. Xcn1 was produced in *X. nematophila* naturally with the help of positive regulatory factors such as FliZ^17^, Hfq^18^ and Lrp^19^. The yield of Xcn1 was increased by deleting or silencing genes of negative regulatory factors such as CpxR^20^, LeuO^19^ and OmpR^21^. In situ product removal method was used to increase the production of Xcn1^22^. Moreover, there were effective tools that block degradation pathways and promoter engineering were employed, which had led to a yield increase of Xcn1 exceeding ten times^23,24^.

Response surface methodology (RSM) was a powerful methodology that was routinely applied in the optimization of bioprocessing of numerous substances such as enzymes (xylanases^25^ and proteases^26^), pecticoligosaccharides^27^, and biodegradation of antibiotics^28^. The medium composition of *X. nematophila* and *X. stockiae* were optimized by response surface methodology to enhancing the antifungal activity of cell-free broth^29,30^. Our previous research showed that antimicrobial activity of cell-free broth was enhanced by manipulating pH^31^, dissolved oxygen^3^ and other fermentation process^32^.

Precursors were crucial factors to improving the production of natural products in the fermentation process^33,34^. The production of antibiotics was increased by adding appropriate precursors. For instance, addition of valine, a biosynthetic precursors of glycopeptide antibiotic A40926, to minimal medium increased A40926 production^35^. Sodium decanoate was an effective precursor for synthesis of daptomycin from Streptomyces roseosporus NRRL11379 which was increased the daptomycin production remarkably^36^. There was no precedent that introducing precursor chemicals for secondary metabolites into medium promotes secondary metabolite production in *X. nematophila*. From the molecular structure of Xcn1, arginine, leucine, urea and acetic acid were identified as precursors of Xcn1 biosynthesis^10,37^.

In order to obtain higher production of Xcn1, composition and their concentrations of the fermentation medium were optimized by RSM in this research. Furthermore, detailed optimization was carried out on parameters such as temperature, pH, fermentation period, seed quantity, broth loading, and shaking speed in the fermentation process. Moreover, several precursor chemicals were investigated to test their effectiveness for the improvement of Xcn1 yield.

## Materials and methods

### Strains and culture conditions

*Xenorhabdus nematophila* YL001 was isolated from entomopathogenic nematode *Steinernema* sp. YL001 which was obtained from Yangling, China^3^. It was preserved at − 78 °C in Shaanxi Research Center of Biopesticide Engineering & Technology. The 16S rRNA gene sequence of *X. nematophila* YL001 obtained accession number EU124381 (https://www.ncbi.nlm.nih.gov/nuccore/EU124381.1) after molecular identification^38^. For Xcn1 production, *X. nematophila* YL001 was maintained in primary variant on NBTA medium, distinguished by absorbing bromothymol blue, and used throughout the study.

A single colony of *X. nematophila* YL001 was transferred into a 250 mL flask containing 100 mL of Luria–Bertani (LB) at 28 °C and 180 rpm for 24 h to seed cultivation. Then, 10 mL of the seed culture was transferred into a 250 mL flask containing 100 mL of fermentation medium and the system was incubated for 48 h with continuous agitation in the constant temperature cultivation oscillator (ZHWY-2112B, Shanghai Zhicheng, China). Aforesaid fermenting procedure was used in all subsequent experiments. The initial fermentation medium, Tryptic Soy Broth (TSB), was composed of the following: 17 g/L Bacto Tryptone, 3 g/L Soy Peptone, 2.5 g/L Glucose, 2.5 g/L K_2_HPO_4_, and 5 g/L NaCl in water; pH 7.2.

### Purification and identification of Xcn1

McInerney’s method was improved and used for isolating and purifying Xcn1^7^. In brief, fermentation broth of *X. nematophila* YL001 was centrifuged to obtain cell-free supernatant. 90% of the water was removed by rotary evaporator and then the concentrated supernatant was extracted with petroleum ether, chloroform and ethyl acetate. The aqueous phase was acidified to pH 3.0 with oxalic acid, subsequently the aqueous phase pH was adjusted to 7.0 with sodium hydroxide. Centrifuge again, and then the supernatant was added to the X-5 macroporous resin column (100 cm × 98 cm, Nankai University Chemical Factory, China) and stood for 6 h for adsorption. Next, the column was eluted with water, 25% methanol (v/v), 50% methanol (v/v), 30% acetone (v/v) containing 0.1 mol/L HCl and 50% acetone (v/v) containing 0.2 mol/L HCl in turn at a flow rate of 3 mL/min. Acetone-containing eluate was concentrated and its pH was adjusted to 8.0 with sodium hydroxide and subsequently loaded onto a 110 cation exchange chromatography column (Tianjin Guangfu Fine 195 Chemical Research Institute, Chian) at a flow rate of 5 mL/min, then eluted with 0.2 mol/L NH_4_Cl. Further purification was conducted by the Sephadex G25 column with 0.1 mol/L NH_4_Cl eluted. Eventually, eluate was adsorbed by X-5 macroporous resin column and eluted by acetone to obtaining purified Xcn1. ^1^H NMR and ^13^C NMR were identified by the nuclear magnetic resonance spectrometer (AVANCE III, 500 MHz, Bruker, Switzerland) (Figs. S1, S2). The structure of Xcn1 was determined by high resolution liquid chromatography mass spectrometry (LC-30A + TripleTOF5600 + , AB SCIEX, USA) (Fig. S3).

### One factor at a time approach (OFAT)

To selecting the original medium with the highest yield of Xcn1, ten commonly used bacterial media were investigated. The fermentation medium included LB medium, TSB medium, Beef peptone yeast (BPY) medium (10 g/L Peptone, 5 g/L Beef Extract, 5 g/L Yeast Extract, 5 g/L Glucose, and 5 g/L NaCl in water; pH 7.2), BR medium (10 g/L Beef Peptone, 3 g/L Beef Extract, and 5 g/L NaCl in water; pH 7.2), KB medium (5 g/L Peptone, 3 g/L Yeast Extract, and 2.5 g/L Glucose in water; pH 7.2), NB medium (5 g/L Beef Extract, 6 g/L Peptone, and 15 g/L NaCl in water; pH 7.2), NB + medium (3 g/L Beef Extract, 5 g/L Peptone, and 10 g/L NaCl in water; pH 7.2), PP3 medium (20 g/L Proteose Peptone in water; pH 7.2), PP3 + medium (20 g/L Proteose Peptone and 10 g/L NaCl in water; pH 7.2), YS medium (5 g/L Yeast Extract, 0.5 g/L (NH_4_)_2_SO_4_, 0.5 g/L K_2_HPO_4_, 0.2 g/L MgSO_4_·7H_2_O, and 5 g/L NaCl in water; pH 7.2).

In the content of 5 g/L, various carbon sources such as glucose, fructose, maltose, starch, dextrin, lactose and sucrose were tested individually. To screen nitrogen sources, 20 g/L concentrations of soy peptone, bacto-tryptone, proteose peptone, beef peptone, yeast extract, potassium nitrate, urea and beef extract were evaluated respectively. To analyze the mineral sources, the inorganic salt such as, magnesium sulfate, sodium chloride, dipotassium hydrogen phosphate and sodium sulfate were tested with content of 10 g/L separately.

The initial pH was set at 5.0, 6.0, 7.0, 8.0 and 9.0. Liquid loading of broth in the 250 mL flask were set at 50, 75, 100, 125 and 150 mL. Seed quantity was set at 4%, 6%, 8%, 10% and 12%. The rotating speeds of the shaker were set at 50, 100, 150 and 200 rpm. Fermentation period were set at 24, 48, 72, 96 and 120 h. And temperatures of fermentation were set at 15, 20, 25, 30 and 35 °C. Several precursors such as arginine, leucine, acetic acid and urea were added to the medium and theirs concentrations were set at 2, 3 and 4 mmol/L. In addition, precursors were added in different time, such as the 0 h, 6 h, 12 h, 18 h, 24 h and 30 h, during fermentation.

### Experimental design and optimization by RSM

The fermentation medium was optimized by response surface methodology. In order to enhance the production of Xcn1, central composite design (CCD) was used to optimize the concentrations of the variables such as carbon source(X_1_), nitrogen source(X_2_) and mineral source(X_3_) with the help of Design expert 10 (Stat-Ease, USA). Variables were analyzed at five different levels (− α, − 1, 0, + 1, and + α). Twenty experimental trials have been carried out for the selected three variables (maltose, proteose peptone and K_2_HPO_4_). These twenty trials included six centre points, six axial points and eight factorial points. After which, the mean value of the response Y (the yield of Xcn1) was assayed in triplicate analysis. The CCD experiment was not only employed to analyze the interactions among the important three selected variables (proteose peptone, maltose and K_2_HPO_4_) but also to find their optimum levels.

### Xcn1 contents analysis by high performance liquid chromatography (HPLC)

Previously purified Xcn1 was seen as the pure product to prepare the standard stock solution with a concentration of 2000 μg/mL. Then, the mother liquor was dissolved with ultra-pure water and diluted it to 1000 μg/mL, 800 μg/mL, 600 μg/mL, 400 μg/mL, 200 μg/mL and 100 μg/mL standard solution. Standard curve, represents the linear relationship between peak area and the concentration of Xcn1, was drawn with Xcn1 concentration as abscissa and absorption peak area as ordinate. The linear regression equation was showed in Eq. (1) and the correlation coefficient was R^2^ = 0.9993 (Fig. S4).

1 $$\text{Y}=4.5173\text{X}+18.658$$

The broth of *X. nematophila* YL001 was centrifuged and the supernatant was added to the activated X-5 macroporous resin column for chromatographic separation. The macroporous resin column was eluted in five times of the volume with deionized-water, 50% methanol (v/v), 30% acetone (v/v) with 0.01 mol/L HCl and 50% acetone (v/v) with 0.02 mol/L HCl successively. The eluent of 30% acetone (v/v) with 0.01 mol/L HCl was collected and dried. Dried samples were dissolved in 10 mL 50% methanol for HPLC detection. Twenty microliters of sample were injected into a reversed-phase C_18_ column (2.1 × 150 mm), eluted with 30% acetonitrile (v/v) in 0.1% trifluoroacetic acid at a flow rate of 1 mL/min, monitored at 312 nm, and the temperature of the column was 25 °C^7^.

### Statistical analysis

Design expert 10 (Stat-Ease, USA) was used to analyze the variables. The 3D response graph and profile for predicted values and desirability level for factors were plotted using the same software. The statistical analysis of the model was performed in the form of analysis of variance (ANOVA). For each variable, the quadratic models were represented as contour plots. In addition, the fermentation conditions and precursors were analyzed using Origin 9.1 and Microsoft office Excel. Data were given from 3 replications ± SE and data with different letters were significantly different at *P* < *0.05* by Duncan’s multiple range test.

## Results

### Identification of Xcn1

**Figure 1:**
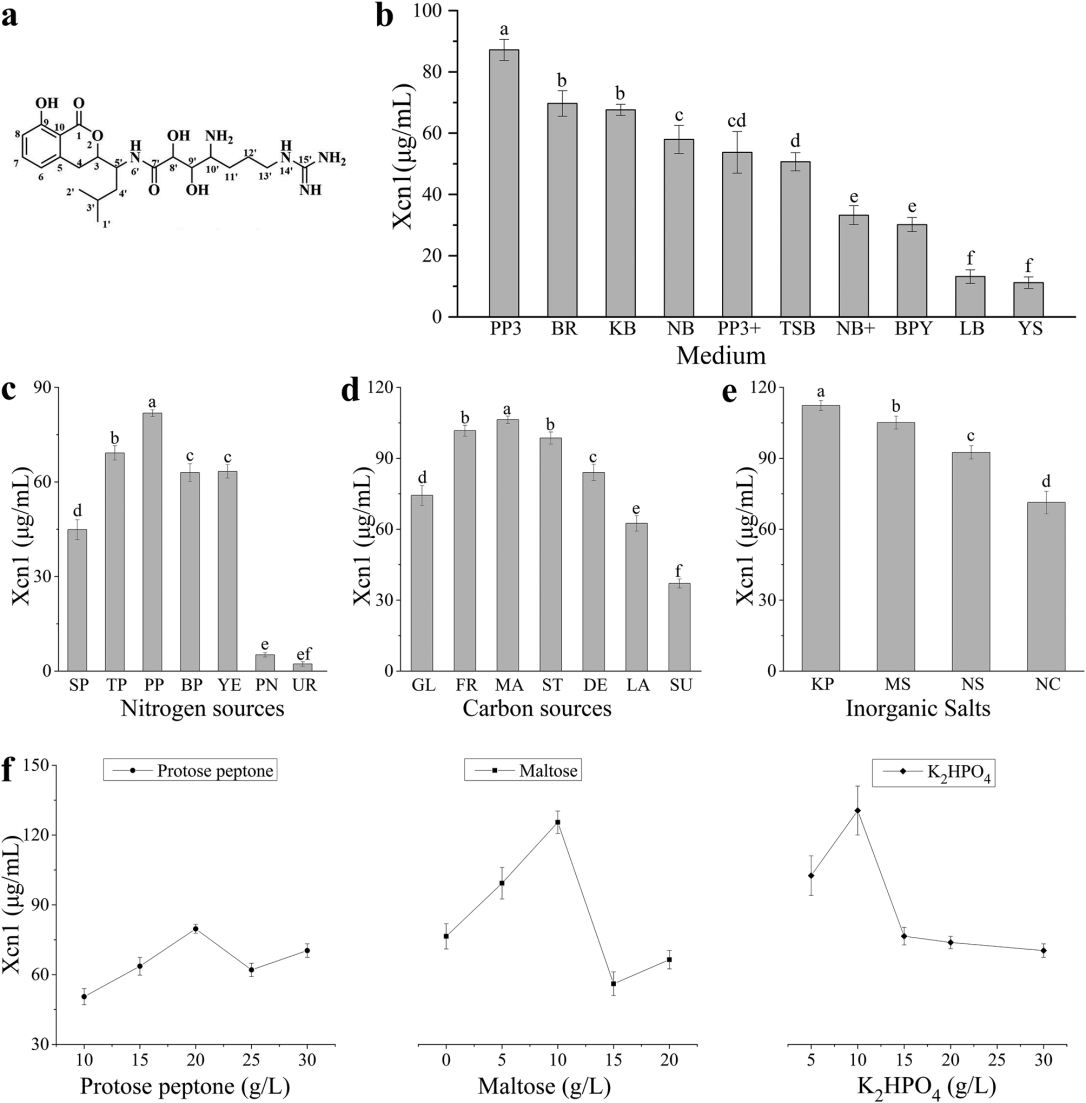
Effects of different medium and nutrient sources on Xcn1 production. (**a**) The structure of Xenocoumacin 1 (Xcn1). (**b**) Effects of different media on Xcn1 production. (**c**) Effects of various nitrogen sources on Xcn1 production. Abbreviation of nitrogen sources (SP: Soy peptone; TP: Tryptone; PP: Proteose peptone; BP: Beef peptone; YE: Yeast extract; PN: Potassium nitrate; UR: Urea; BA: Beef paste). (**d**) Effects of different carbon sources on Xcn1 production. Abbreviation of carbon sources (GL: Glucose; FR: Fructose; MA: Maltose; ST: Starch; DE: Dextrin; LA: Lactose; SU: Sucrose). (**e**) Effects of various inorganic salts on Xcn1 production. Abbreviation of inorganic salts (KP: K_2_HPO_4_; MS: MgSO_4_; NS: Na_2_SO_4_; NC: NaCl). (**f**) Effects of various concentrations of proteose peptone, maltose and K_2_HPO_4_ on Xcn1 production. The data represent the mean values of three independent replicates, and the error bars represent the standard deviations. Data with different letters are significantly different at *P* < *0.05* by Duncan’s multiple range tests.

The chemical structure of Xcn1 was showed in Fig. 1a. The signals of hydrogen atoms and carbon atoms of Xcn1 were well presented in the nuclear magnetic spectrum, and their chemical shifts and coupling constants are also consistent with the reported data^7^. The data analysis of ^1^H NMR and ^13^C NMR spectra for Xcn1 were showed in Table S1.

### Effect of different media on Xcn1 production

The results of Xcn1 production of *X. nematophila* YL001 in different media were shown in Fig. 1b and Fig. S5. The results indicated that PP3 medium showed a maximum Xcn1 production (87.21 μg/mL), followed by BR medium (69.71 μg/mL) and KB medium (67.61 μg/mL). Lower Xcn1 production was observed in YS medium (11.12 μg/mL).

### Effect of various nutrient sources on Xcn1 production

Among various nitrogen sources mentioned in Fig. 1c and Fig. S6, the *X. nematophila* YL001 produced the maximum Xcn1 in proteose peptone (81.82 μg/mL), followed by tryptone (69.19 μg/mL) and yeast extract (63.34 μg/mL). Among carbon source, maltose showed a maximum Xcn1 production (106.31 μg/mL) followed by fructose (101.67 μg/mL) and starch (98.57 μg/mL). Lower Xcn1 production was observed with the sucrose (37.06 μg/mL) (Fig. 1d and Fig. S7). Among the various inorganic salt studied (Fig. 1e and Fig. S8), the *X. nematophila* YL001 produced the maximum Xcn1 (112.30 μg/mL) in K_2_HPO_4_ and the minimum level of Xcn1 (71.30 μg/mL) in NaCl. Proteose peptone, maltose and K_2_HPO_4_ were chosen as the source of nitrogen, carbon, and inorganic salt for further experiments (Fig. 1f and Fig. S9).

### Optimization of medium constituents by response surface method

After determination of nitrogen sources, carbon sources and inorganic salts in the medium, the combined effects of concentration of medium constituents on Xcn1 production were further investigated. Table 1 showed experimental range and levels of the independent variables. The experimental results listed in Table 2 were analyzed using multiple regressions. The production of Xcn1 was maximum at run 11 (113.65 μg/mL). The second-order polynomial model was proposed to evaluate the optimum levels of these selected variables and was shown in Eq. (2).

**Table 1:** Experimental range and levels of the independent variables through central composite design.

Variables	Factors	Range and levels				
		− 1.682	− 1	0	1	1.682
${X}_{1}$	Maltose (g/L)	4.95	7	10	13	15.05
${X}_{2}$	Proteose peptone (g/L)	9.91	14	20	26	30.09
${X}_{3}$	K_2_HPO_4_ (g/L)	2.48	3.5	5	6.5	7.52

Converting the coded values of variables to the actual dose of nutrients using the following formula: ${X}_{1}$= (Maltose—10)/3; ${X}_{2}$= (Proteose peptone—20)/6; ${X}_{3}$= (K_2_HPO_4_—5)/1.5

**Table 2:** Actual doses of nutrient sources set by central composite design and predicted production of Xcn1 from *Xenorhabdus nematophila* YL001.

Run	Maltose(g/L)	Proteose peptone(g/L)	K_2_HPO_4_(g/L)	Xcn1 (μg/mL)
1	10	9.91	5	34.79
2	10	20	5	108.28
3	10	30.09	5	75.16
4	7	26	3.5	99.16
5	13	26	6.5	41.45
6	7	14	6.5	56.66
7	10	20	5	103.94
8	10	20	7.52	62.52
9	10	20	5	103.42
10	10	20	5	105.41
11	10	20	2.48	113.65
12	7	26	6.5	77.84
13	10	20	5	104.72
14	13	14	6.5	63.56
15	15.05	20	5	53.32
16	13	26	3.5	72.57
17	7	14	3.5	56.66
18	13	14	3.5	63.56
19	4.95	20	5	101.25
20	10	20	5	110.23

2 $$\text{Y}=-469.45715+33.78184{X}_{1}+31.64599{X}_{2}+43.41016{X}_{3}-0.53319{X}_{1}{X}_{2}-0.27222{X}_{1}{X}_{3}-0.72833{X}_{2}{X}_{3}-1.24623{{X}_{1}}^{2}-0.53066{{X}_{2}}^{2}-3.28788{{X}_{3}}^{2}$$

**Figure 2:**
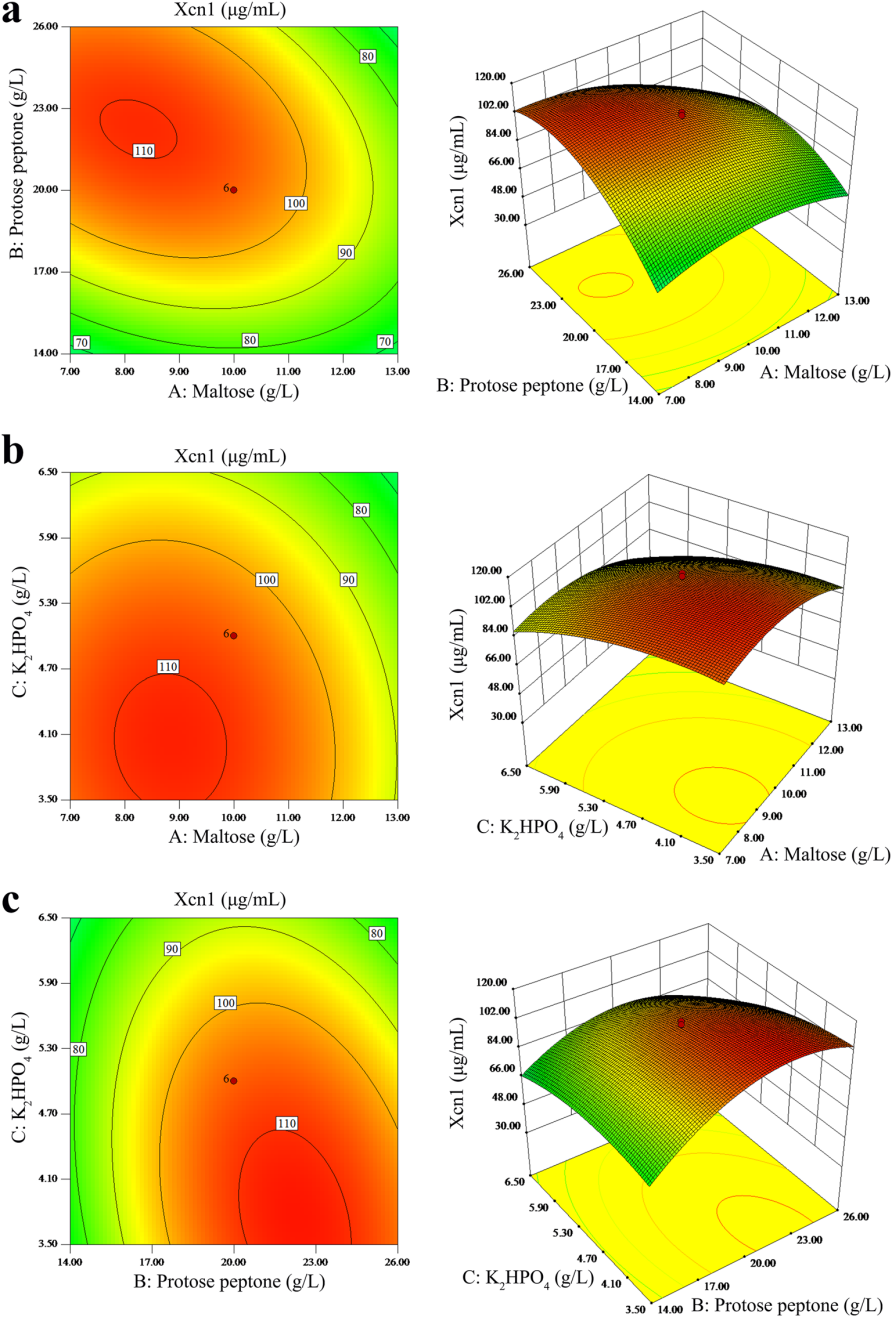
Response surface plot and contour plot. (**a**) The combined effects of maltose and proteose peptone on the Xcn1 production. (**b**) The combined effects of maltose and K_2_HPO_4_ on the Xcn1 production. (**c**) The combined effects of proteose peptone and K_2_HPO_4_ on the Xcn1 production.

In this study, the determination coefficient (R^2^) confirmed the importance of statistical design, exhibiting minor experimental error and a fit regression equation. The R^2^ of this designed CCD model was 0.9443. The F value of the model is 18.38, while the *P* value is less than 0.0001, indicating that the model has significance (Table 3). The saddle or elliptical nature of contour plot showed the significance of the good interactions between the respective variables. Figure 2 showed the contour plot and 3D response surface plot for the Xcn1 yield generated by the predicted CCD model. According to the model equation, we predicted that a maximum Xcn1 yield of 113.65 μg/mL could be achieved at 12.74 g/L maltose, 20.83 g/L proteose peptone and 3.77 g/L K_2_HPO_4_. By HPLC, the content of Xcn1 was 117.99 μg/mL with the optimized medium, which was close to the predicted response (Table 2 and Fig. S10). Compared with the output of Xcn1 of TSB medium (50.67 μg/mL), the output of Xcn1 of the optimized PP3 medium (113.65 μg/mL) increased by 112.65%.

**Table 3:** Evaluation of the prediction model of multiples linear regression for Xcn1 production.

Source	Sum of Squares	Df	Mean Square	F Value	*P* Value
Model	11599.14	9	1288.79	18.38	< 0.0001**
A- Maltose (${\text{X}}_{1}$)	1233.45	1	1233.45	17.59	0.0018
B- Proteose peptone (${\text{X}}_{2}$)	1027.77	1	1027.77	14.66	0.0033
C- K_2_HPO_4_ (${\text{X}}_{3}$)	1403.17	1	1403.17	20.01	0.0012
AB	736.90	1	736.90	10.51	0.0088
AC	12.00	1	12.00	0.17	0.6878
BC	343.74	1	343.74	4.90	0.0512
A^2^	1812.96	1	1812.96	25.85	0.0005
B^2^	5259.52	1	5259.52	75.00	< 0.0001
C^2^	788.68	1	788.68	11.25	0.0073
Residual	701.28	10	70.13		
Lack of Fit	665.31	5	133.06	18.49	0.0031**
Pure Error	35.98	5	7.20		
Cor Total	12300.42	19			

R^2^ = 94.43%, C.V. % = 10.41.

**Significant at 1% level.

### Optimization of cultural conditions

**Figure 3:**
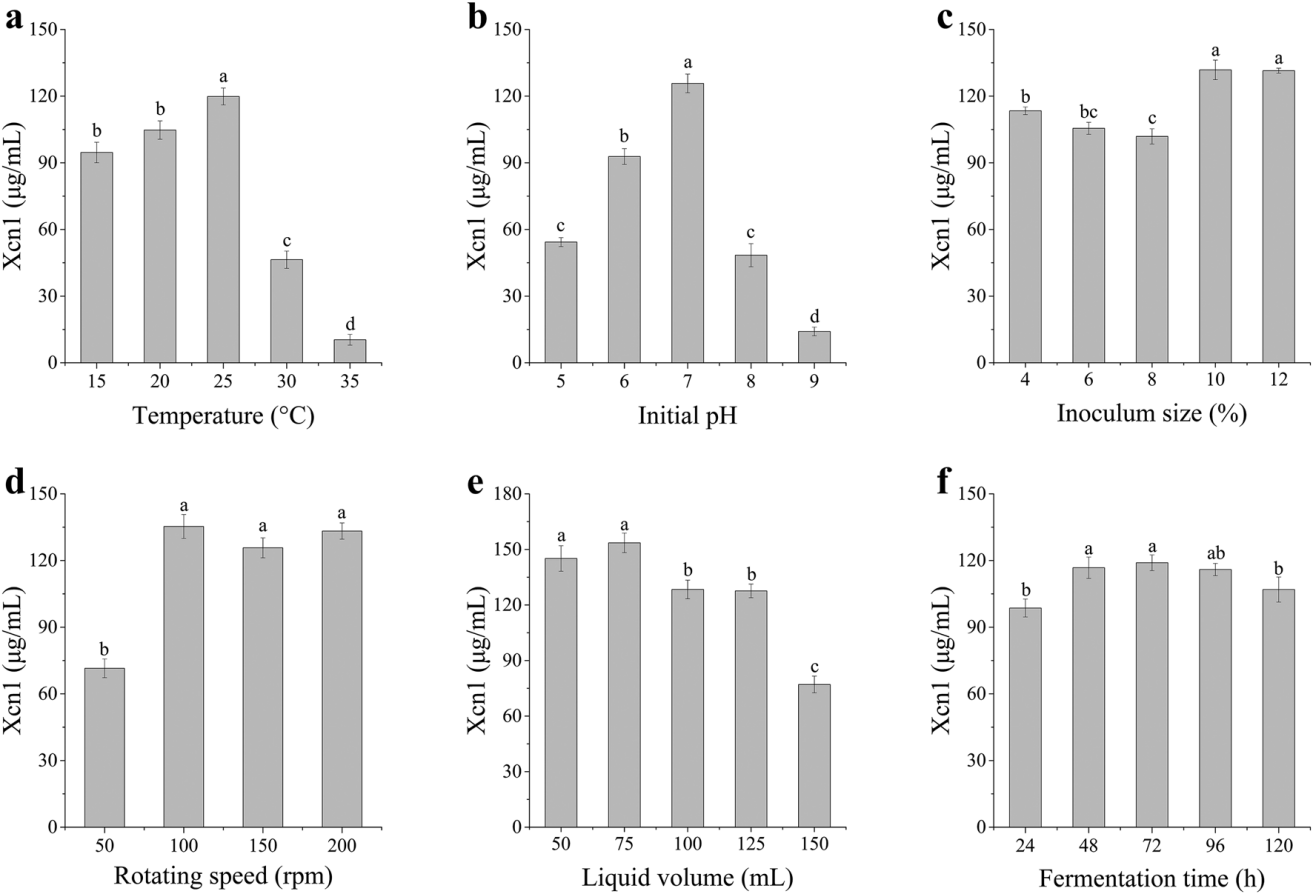
Effects of different fermentation conditions on the production of Xcn1. (**a**) Effects of various fermentation temperatures on Xcn1 production. (**b**) Effects of different initial pH of fermentation medium on Xcn1 production. (**c**) Effects of various inoculum size on Xcn1 production. (**d**) Effects of different rotating speed of shaker on Xcn1 production. (**e**) Effects of various volume of liquid medium on Xcn1 production. (**f**) Effects of different fermentation time on Xcn1 production. The data represent the mean values of three independent replicates, and the error bars represent the standard deviations. Data with different letters are significantly different at *P* < *0.05* by Duncan’s multiple range tests.

The production of Xcn1 reached the optimal yeild at 25 °C (Fig. 3a and Fig. S11). It was selected that pH 7 for optimal production (125.72 μg/mL) of Xcn1 (Fig. 3b and Fig. S11). 10% was selected as the optimal inoculum size (Fig. 3c and Fig. S12). 100 rpm was selected as the optimal rotating speed (Fig. 3d and Fig. S12). It showed that the optimal medium loading was 75 mL medium in a 250 mL flask (Fig. 3e and Fig. S13). There was no significant increase in the production of Xcn1 when the fermentation time increased after 48 h (Fig. 3f and Fig. S13). Consequently, 48 h was selected as the optimal fermentation time.

### Effect of precursors on Xcn1 production

**Figure 4:**
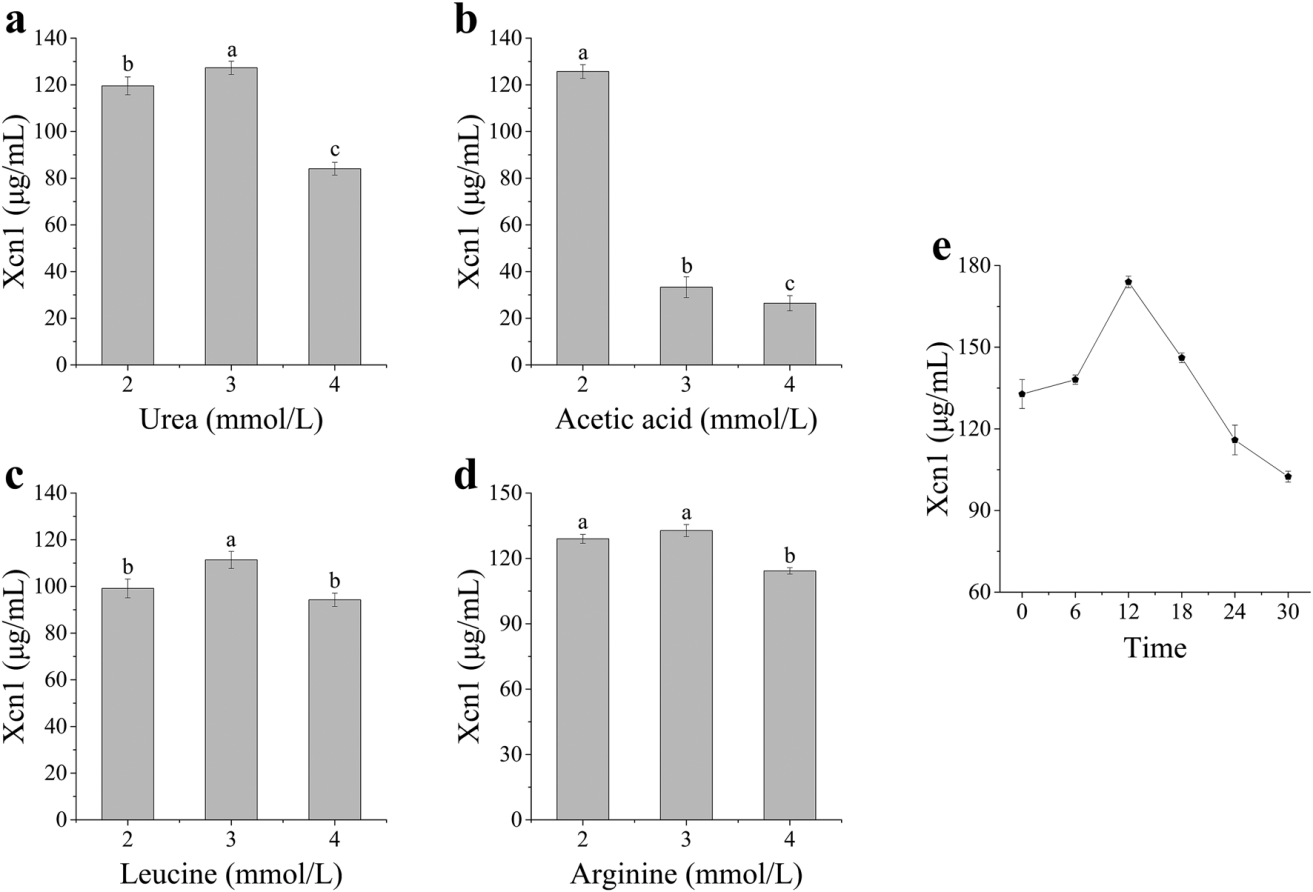
Effects of different precursor substances concentration and arginine adding time on Xcn1 production. (**a**) Effects of various concentration of urea on Xcn1 production. (**b**) Effects of different concentration of acetic acid on Xcn1 production. (**c**) Effects of various concentration of leucine on Xcn1 production. (**d**) Effects of different concentration of arginine on Xcn1 production. (**e**) Effects of adding time of arginine on Xcn1 production. The data represent the mean values of three independent replicates, and the error bars represent the standard deviations. Data with different letters are significantly different at *P* < *0.05* by Duncan’s multiple range tests.

Markedly, in those tested precursor, arginine was the best for Xcn1 production and its optimal concentration was 3 mmol/L (Fig. 4 and Fig. S14–15).The production of Xcn1 was assessed at 48 h and reached the maximum yield when arginine was added to the medium at the 12 h (Fig. 4e and Fig. S16). With the optimal concentration (3 mmol/L), arginine was added to the fermentation medium at the 12 h.

### Discussions

Medium played a decisive role in the whole fermentation process of microorganisms^30^. By optimizing the nutrition of medium, the yield of Xcn1 was enhanced from 50.73 μg/mL to 117.99 μg/mL. Proteose peptone was the most suitable nitrogen sources for Xcn1 production. Previous studies showed that *X. nematophila* YL001 produced the most antibiotic activity when proteose peptone was used as nitrogen source^30^. Going further, it indicated that Xcn1 was the main active component of the antibiotic produced by *X. nematophila* YL001^10,39^. In order to verify the accuracy of prediction by RSM, obtained medium formula was used to flask fermentation. The measured yield of Xcn1 was consisted with the predicted result so that RSM was an effective method for optimizing media for antibiotic production.

To ensure accuracy, the factors for RSM optimization were usually limited to 4. Accordingly, optimization of 6 fermentation conditions was carried out by one factor at a time approach. Through the optimization of fermentation conditions, the yield of Xcn1 reached 153.56 μg/mL. Temperature and pH had significant influence on Xcn1 production of *X. nematophila* YL001, and high or low temperature and pH both led to a sharp decrease in Xcn1 production (Fig. 4). Several other environmental conditions, such as inoculum size, shaking speed, broth loading and fermentation time, could not make such a huge change in Xcn1 production. In this study, 7.0 was the most suitable pH for Xcn1 production. However, this was not consistent with the results obtained in previous studies. The weak alkaline pH environment was found to be beneficial for the production of Xcn1 and the production of Xcn1 were 2.49-fold higher at pH 8.5 relative to that at pH 7.0 in *X. nematophila* YL001^2^. To some extent, it may be due to the combined effects of various conditions of fermentation, such as fermentation time and broth loading, on Xcn1 production. For example, previous study showed that 50 mL medium was loaded in a 250 mL flask and incubated for 72 h, which resulted 8.5 was a better pH^2^. Accordingly, more precise control of pH and temperature would help to increase the yield of Xcn1. The optimal medium and fermentation process were chosen for this study in an effort to enhance the production of Xcn1 in *X. nematophila* YL001.

Four precursors were involved in the biosynthesis of Xcn1 directly or indirectly. The synthesis of arginine by microorganism needed urea to provide nitrogen. Arginyl thioester was condensed with N-acyl-D-asparaginyl thioetser into the long chain of Xcn1, which occurred in the initial stage of Xcn1 long chain biosynthesis^10^. Hence, arginine was the most helpful precursor for Xcn1 biosynthesis, which is consistent with the results of our study. Moreover, leucine and acetic acid were involved in the long chain synthesis of Xcn1, respectively, to form peptide bonds and extend the carbon chain^10^. Arginine was added to the medium to increase the production of Xcn1 in this study. If precursors, such as arginine, leucine and acetic acid, were added in the media with proper proportion at the right time, the production of Xcn1 would be further improved.

Previous studies evaluated the content of antibacterial active substances in fermentation broth of *X. nematophila* YL001 according to the size of bacteriostatic circle which was not accurate enough^30^. In this study, HPLC method was provided a more precise and accurate method for the Xcn1 detection and was proved to be a powerful tool for the optimization Xcn1 production by *X. nematophila* YL001.

In an industrial point of view, to improve the production, various methods and techniques should be screened for Xcn1 production. A series of published research suggested that fermentation optimization had limited effect on improving Xcn1 production compared to modifying the genome of *X. nematophila*^23,24^. However, fermentation optimization was still an indispensable part of industrial production. Furthermore, the fermentation of *X. nematophila* YL001 would be studied in a fermentation facility to further optimize the fermentation conditions at pilot sacle, so as to improve the output of Xcn1 and lay a foundation for its industrial production.

## Conclusions

Medium, fermentation conditions and precursors were regulated and used to enhance the production of Xcn1. The production of Xcn1 on the optimal fermentation conditions reached 173.99 μg/mL, which was 3.43 times as much as that on the original conditions (TSB medium). The results will be helpful for the development of *X. nematophila* YL001 cultivation process for efficient Xcn1 production and lay a foundation for its industrial production, and promotes the application of Xcn1 in sustainable agricultural development.

### Supplementary Information

Supplementary Information.

## Data Availability

All data generated or analyzed during this study are included in this published article and Supplementary materials.
