# Supplementary material for: Enhancing the yield of Xenocoumacin 1 in Xenorhabdus nematophila YL001 by optimizing the fermentation process
Source: Sci Rep. 2024 Jun 12;14:13506. doi: 10.1038/s41598-024-63794-2 (PMC11169479; doi:10.1038/s41598-024-63794-2)
Supplement: Supplementary file 1 — Supplementary Information. [file 41598_2024_63794_MOESM1_ESM.docx]

Enhancing the yield of Xenocoumacin 1 in *Xenorhabdus nematophila* YL001 by optimizing the fermentation process

**Yunfei Han^1, 3^, Shujing Zhang^1, 2^, Yang Wang^1, 3^, Jiangtao Gao^1, 3^, Jinhua Han^1, 3^, Zhiqiang Yan^1, 3^, Yongquan Ta^1, 3^, Yonghong Wang^1,3#^**

^1^ Key Laboratory of Plant Protection Resources and Pest Management, Ministry of Education, College of Plant Protection, Northwest A&F University, 22 Xinong Road, Yangling 712100, Shaanxi, China

^2^ Key Laboratory of Green Prevention and Control of Tropical Plant Diseases and Pests, Ministry of Education, School of Tropical Agriculture and Forestry, Hainan University, 58 People's Avenue, Haikou 570228, Hainan, China

^3^ Shaanxi Research Center of Biopesticide Engineering & Technology, College of Plant Protection, Northwest A&F University, 22 Xinong Road, Yangling 712100, Shaanxi, China

**A list of supplementary figures**

Fig. S1 ^1^H Nuclear magnetic resonance spectrum of Xenocoumacin 1 (Xcn1).

Fig. S2 ^13^C Nuclear magnetic resonance spectrum of Xcn1.

Fig. S3 High resolution mass spectrum of Xcn1.

Fig. S4 Standard curve and high performance liquid chromatogram (HPLC) of previously purified Xcn1.

Fig. S5 HPLC of effects of different medium on Xcn1 production.

Fig. S6 HPLC of effects of different nitrogen sources on Xcn1 production.

Fig. S7 HPLC of effects of different carbon sources on Xcn1 production.

Fig. S8 HPLC of effects of different inorganic salts on Xcn1 production.

Fig. S9 HPLC of effects of various concentration of proteose peptone and maltose on Xcn1 production.

Fig. S10 HPLC of experimental trials of central composite design.

Fig. S11 HPLC of effects of various fermentation temperatures and medium’s initial pH on Xcn1 production.

Fig. S12 HPLC of effects of various inoculum size and rotating speed on Xcn1 production.

Fig. S13 HPLC of effects of various liquid medium volume and fermentation time on Xcn1 production.

Fig. S14-15 HPLC of effects of different precursor substances and its concentration on Xcn1 production.

Fig. S16 HPLC of effects of different adding time of arginine on Xcn1 production.

^^

**Figure S1 ^1^H Nuclear magnetic resonance spectrum of Xenocoumacin 1 (Xcn1).**

^1^H NMR (500 MHz, D_2_O) δ 7.56 (t, *J* = 7.9 Hz, 1H), 6.94 (d, *J* = 8.5 Hz, 1H), 6.91 (d, *J* = 7.4 Hz, 1H), 4.69-4.79 (m, 1H), 4.33 (d, *J* = 6.0 Hz, 1H), 4.27 (dt, *J* = 9.8, 4.8 Hz 1H), 4.17 (dd, *J* = 6.0, 4.0 Hz, 1H), 3.53 (td, *J* = 9.8, 6.6 Hz 1H), 3.27 (t, *J* = 6.6 Hz, 2H), 3.07-3.10 (m, 2H), 1.95-1.87 (m, 1H), 1.85-1.74 (m, 3H), 1.73-1.66 (m, 1H), 1.66-1.59 (m, 1H), 1.50 (td, *J* = 9.6, 4.7 Hz, 1H), 0.97 (d, *J* = 6.6 Hz, 3H), 0.89 (d, *J* = 6.6 Hz, 3H).

^^

**Figure S2 ^13^C Nuclear magnetic resonance spectrum of Xenocoumacin 1 (Xcn1).**

^13^C NMR (125 MHz, D_2_O) δ 172.50, 169.10, 159.00, 155.67, 138.80, 136.00, 118.18, 114.41, 107.11, 80.34, 71.52, 69.45, 52.49, 48.52, 39.54, 37.44, 28.10, 23.41, 23.33, 23.15, 21.39, 19.60.

**Figure S3 High resolution mass spectrum of Xenocoumacin 1 (Xcn1).**

HRMS (m/z): [M+H]^+^ calculated for C_22_H_36_N_5_O_6_, 466.2660; found, 466.2638.


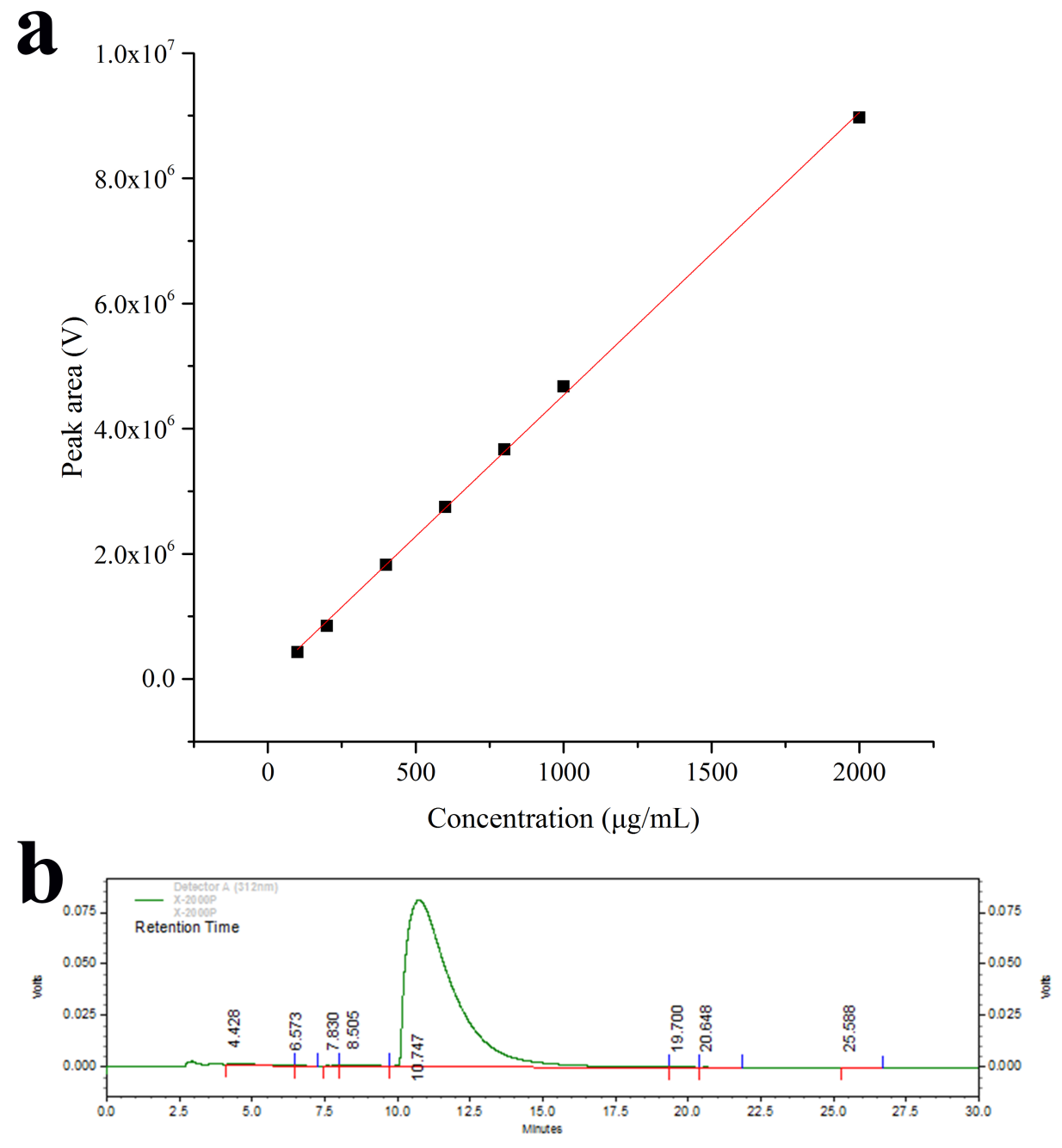


**Figure S4 Standard curve and high performance liquid chromatogram (HPLC) of previously purified Xcn1.**

a Standard curve that describing the relationship between Xcn1 concentration and peak area; Standard equation: y=4.5173x+18.658.

b: High performance liquid chromatogram (HPLC) of previously purified Xcn1. Xcn1 concentration: 200 μg/mL; Retention Time: 10.747 min.


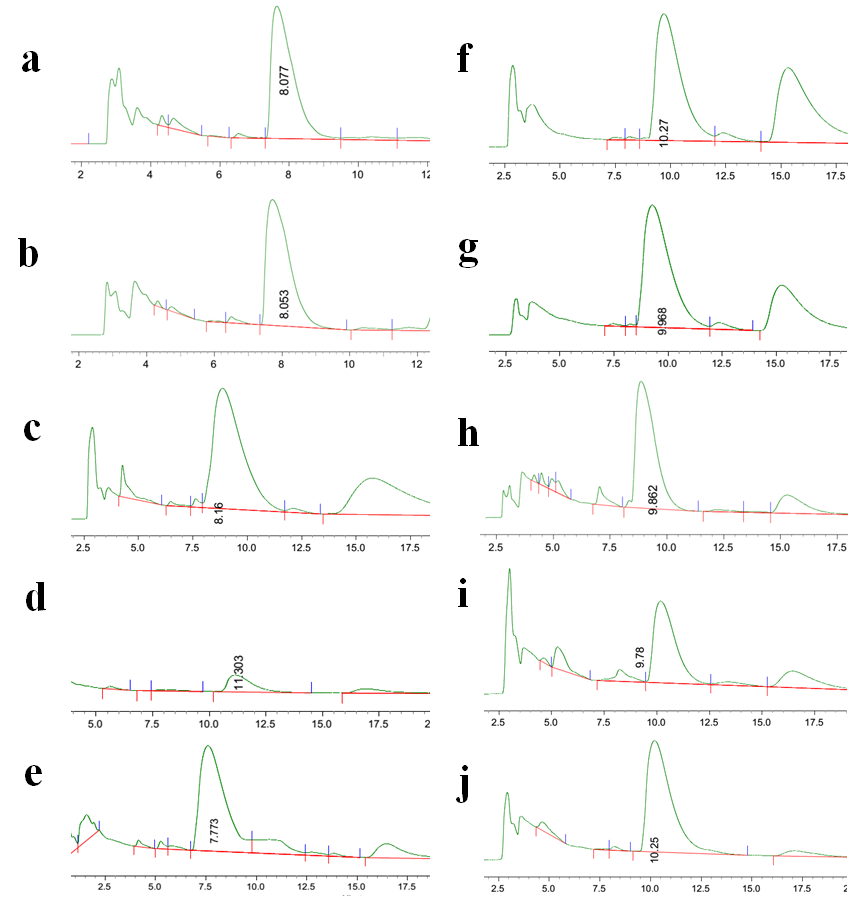


**Fig. S5 HPLC of effects of different medium on Xcn1 production.**

a: Medium: PP3+; Retention Time: 8.077 min; Peak area: 6005433 volts. b: Medium: BR; Retention Time: 8.053 min; Peak area: 9729949 volts. c: Medium: PP3; Retention Time: 8.160 min; Peak area: 12183758 volts. d: Medium: YS; Retention Time: 11.303 min; Peak area: 1879333 volts. e: Medium: TSB; Retention Time: 7.773 min; Peak area: 6847882 volts. f: Medium: NB+; Retention Time: 10.270 min; Peak area: 4669670 volts. g: Medium: NB; Retention Time: 9.968 min; Peak area: 8075262 volts. h: Medium: KB; Retention Time: 9.862 min; Peak area: 9282626 volts. i: Medium: LB; Retention Time: 9.78 min; Peak area: 1703159 volts. j: Medium: BPY; Retention Time: 8.077 min; Peak area: 6005433 volts.


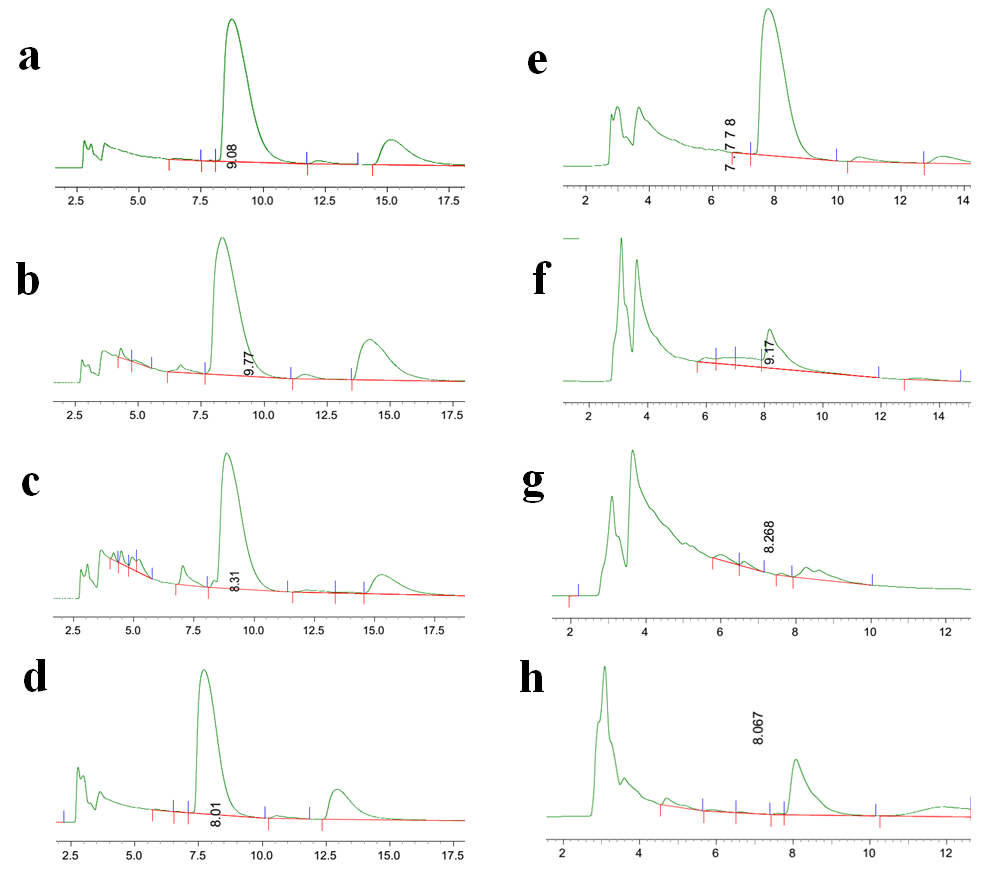


**Fig. S6 HPLC of effects of different nitrogen sources on Xcn1 production.**

a: Nitrogen source: Tryptone; Retention Time: 9.08 min; Peak area: 9194649 volts. b: Nitrogen source: Proteose peptone; Retention Time: 9.77 min; Peak area: 12078498 volts. c: Nitrogen source: Soy peptone; Retention Time: 8.310 min; Peak area: 6118368 volts. d: Nitrogen source: Beef peptone; Retention Time: 8.01 min; Peak area: 8527896 volts. e: Nitrogen source: Yeast extract; Retention Time:7.778 min; Peak area: 8489950 volts. f: Nitrogen source: Urea; Retention Time: 9.17 min; Peak area: 291051 volts. g: Nitrogen source: Beef paste; Retention Time: 8.268 min; Peak area: 167729 volts. h: Nitrogen source: Potassium nitrate; Retention Time: 8.067 min; Peak area: 637980 volts.


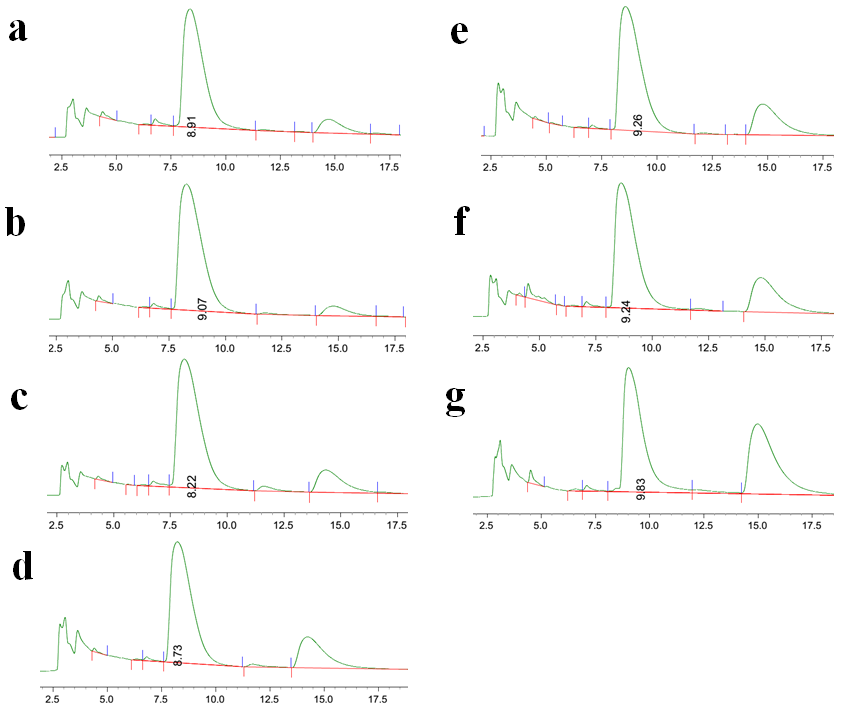


**Fig. S7 HPLC of effects of different carbon sources on Xcn1 production.**

a: Carbon source: Glucose; Retention Time: 8.91 min; Peak area: 9777381 volts. b: Carbon source: Fructose; Retention Time: 9.07 min; Peak area: 13951366 volts. c: Carbon source: Maltose; Retention Time: 8.22 min; Peak area: 14347081 volts. d: Carbon source: Starch; Retention Time: 8.73 min; Peak area: 13529902 volts. e: Carbon source: Dextrin; Retention Time: 9.26 min; Peak area: 11689554 volts. f: Carbon source: Lactose; Retention Time: 9.24 min; Peak area: 9357272 volts. g: Carbon source: Sucrose; Retention Time: 9.83 min; Peak area: 5169735 volts.


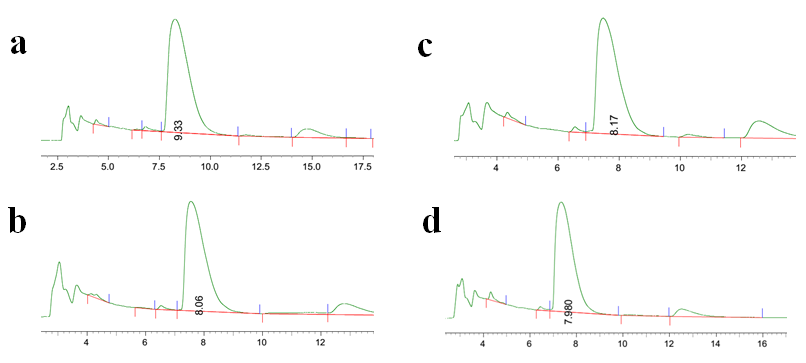


**Fig. S8 HPLC of effects of different inorganic salts on Xcn1 production.**

a: Inorganic salt: MgSO_4_; Retention Time: 9.33 min; Peak area: 9027961 volts. b: Inorganic salt: NaCl; Retention Time: 9.06 min; Peak area: 6743110 volts. c: Inorganic salt: **Na_2_SO_4_**; Retention Time: 8.17 min; Peak area: 8040027 volts. d: Inorganic salt: **KH_2_PO_4_**; Retention Time: 7.980 min; Peak area: 8693229 volts.


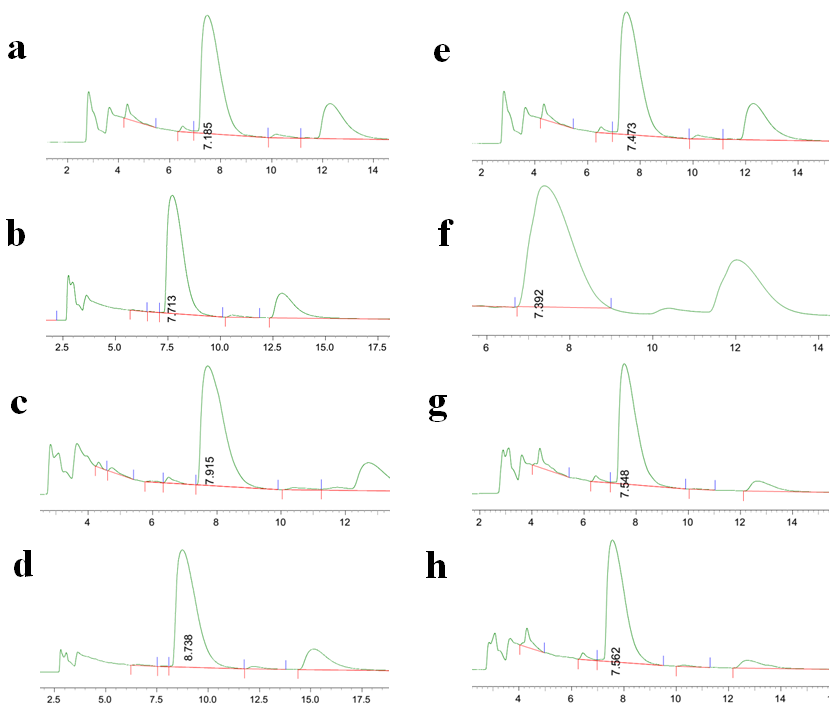


**Fig. S9 HPLC of effects of various concentration of proteose peptone and maltose on Xcn1 production.**

a: Proteose peptone: 10 g/L; Retention Time: 7.185 min; Peak area: 6866884 volts. b: Proteose peptone: 15 g/L; Retention Time: 7.713 min; Peak area: 8642183 volts. c: Proteose peptone: 25 g/L; Retention Time: 7.915 min; Peak area: 8425353 volts. d: Proteose peptone: 30 g/L; Retention Time: 8.738 min; Peak area: 9552419 volts. e: Maltose: 5 g/L; Retention Time: 7.473 min; Peak area: 13475432 volts. f: Maltose: 10 g/L; Retention Time: 7.392 min; Peak area: 17027297 volts. g: Maltose: 15 g/L; Retention Time: 7.548 min; Peak area: 5139646 volts. h: Maltose: 20 g/L; Retention Time: 7.562 min; Peak area: 7615896 volts.


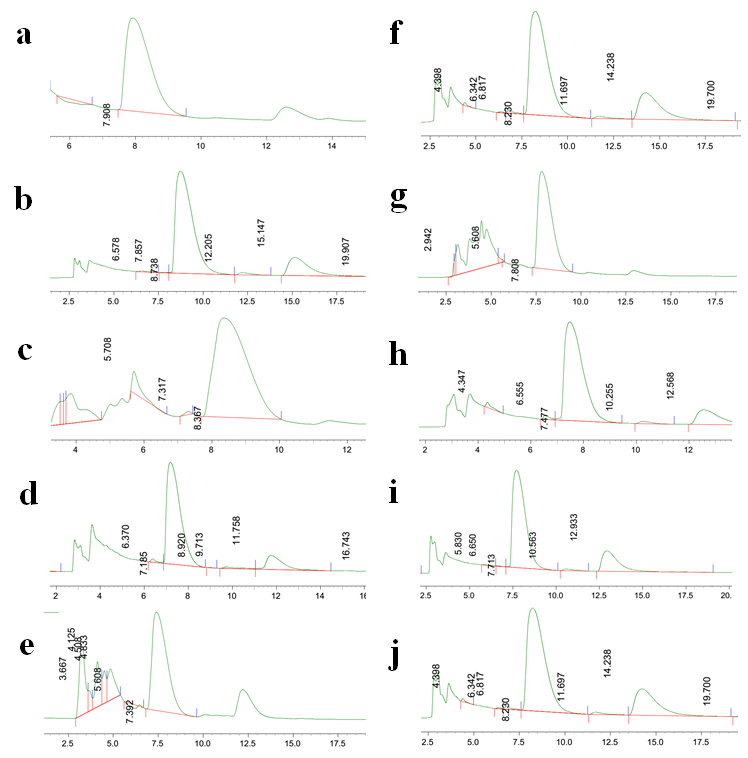


**Fig. S10 HPLC of experimental trials of central composite design.**

a: Run 1; Retention Time: 7.908 min; Peak area: 4733364 volts. b: Run 3; Retention Time: 8.738 min; Peak area: 10204266 volts. c: Run 4; Retention Time: 8.367 min; Peak area: 13456722 volts. d: Run 6; Retention Time: 7.185 min; Peak area: 7697164 volts. e: Run 8; Retention Time: 7.392 min; Peak area: 8491305 volts. f: Run 11; Retention Time: 8.230 min; Peak area: 15420392 volts. g: Run 14; Retention Time: 7.808 min; Peak area: 8632245 volts. h: Run 15; Retention Time: 7.477 min; Peak area: 7244531 volts. i: Run 18; Retention Time: 7.713 min; Peak area: 8632245 volts. j: Run 19; Retention Time: 8.230 min; Peak area: 13739956 volts.


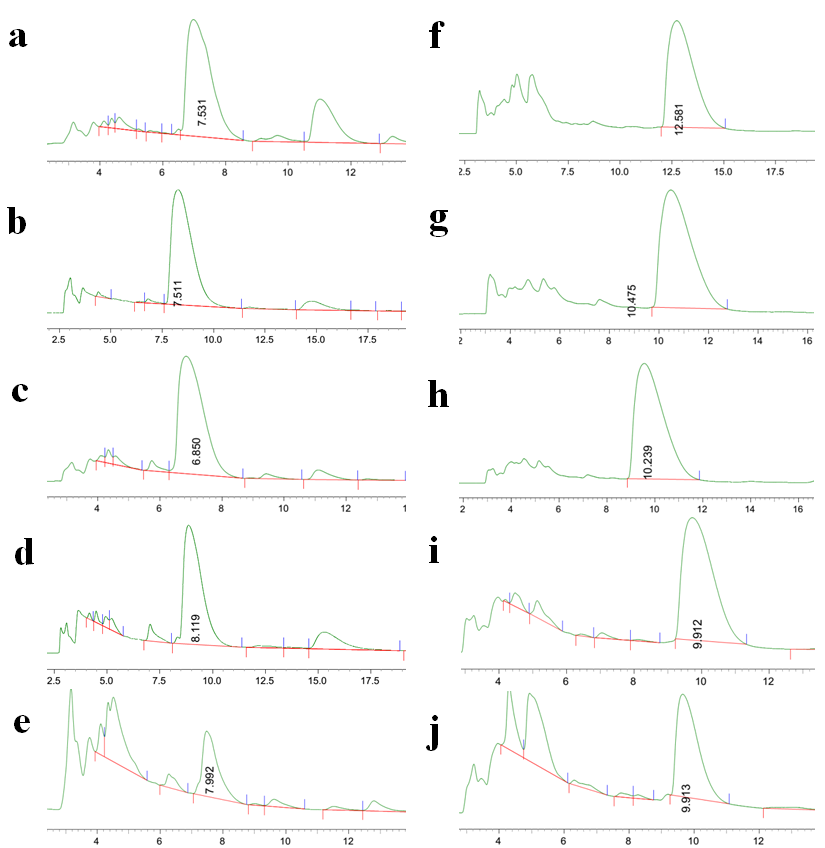


**Fig. S11 HPLC of effects of various fermentation temperatures and medium’s initial pH on Xcn1 production.**

a: Temperature: 15°C; Retention Time: 7.531 min; Peak area: 12697444 volts. b: Temperature: 20°C; Retention Time: 7.511 min; Peak area: 14309634 volts. c: Temperature: 25°C; Retention Time: 6.850 min; Peak area: 16554236 volts. d: Temperature: 30°C; Retention Time: 8.119 min; Peak area: 6409909 volts. e: Temperature: 35°C; Retention Time: 7.992 min; Peak area: 1313432 volts. f: pH 5; Retention Time: 12.581 min; Peak area: 7665630 volts. g: pH 6; Retention Time: 10.475 min; Peak area: 12797996 volts. h: pH 7; Retention Time: 10.239 min; Peak area: 17198164 volts. i: pH 8; Retention Time: 9.912 min; Peak area: 6832296 volts. j: pH 9; Retention Time: 9.913 min; Peak area: 1954800 volts.


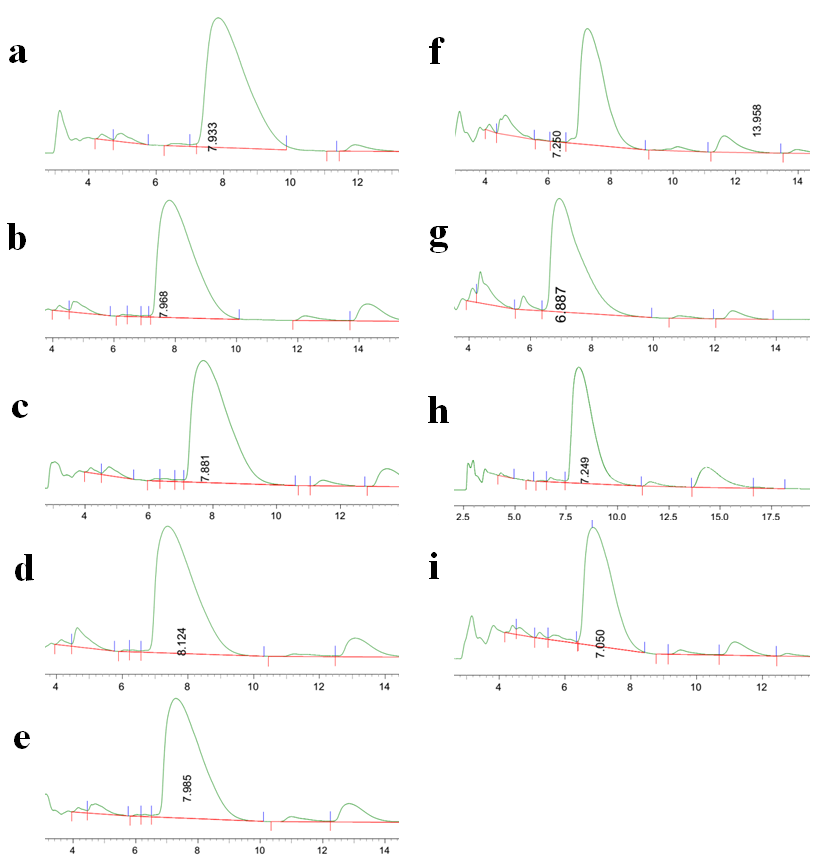


**Fig. S12 HPLC of effects of various inoculum size and rotating speed on Xcn1 production.**

a: Inoculum size: 4%; Retention Time: 7.933 min; Peak area: 15655486 volts. b: Inoculum size: 6%; Retention Time: 7.968 min; Peak area: 14113870 volts. c: Inoculum size: 8%; Retention Time: 7.881 min; Peak area: 13401598 volts. d: Inoculum size: 10%; Retention Time: 8.124 min; Peak area: 18466609 volts. e: Inoculum size: 12%; Retention Time: 7.985 min; Peak area: 17855188 volts. f: Rotating speed: 50 rpm; Retention Time: 7.250 min; Peak area: 19550504 volts. g: Rotating speed: 100 rpm; Retention Time: 6.887 min; Peak area: 17512750 volts. h: Rotating speed: 150 rpm; Retention Time: 7.249 min; Peak area: 17166746 volts. i: Rotating speed: 200 rpm; Retention Time: 7.050 min; Peak area: 18545614 volts.


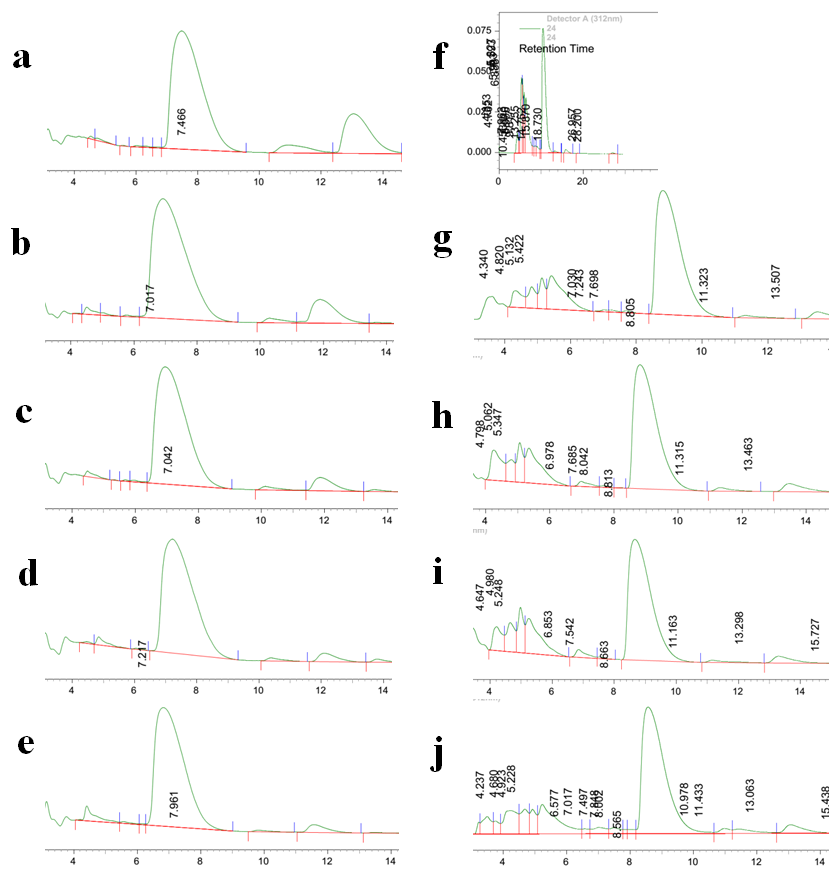


**Fig. S13 HPLC of effects of various liquid medium volume and fermentation time on Xcn1 production.**

a: Volume: 50 mL; Retention Time: 7.466 min; Peak area: 10022568 volts. b: Volume: 75 mL; Retention Time: 7.017 min; Peak area: 20527314 volts. c: Volume: 100 mL; Retention Time: 7.042 min; Peak area: 16799343 volts. d: Volume: 125 mL; Retention Time: 7.217 min; Peak area: 17182542 volts. e: Volume: 150 mL; Retention Time: 7.961 min; Peak area: 10234761 volts. f: Fermentation time: 24 h; Retention Time: 10.453 min; Peak area: 4473431 volts. g: Fermentation time: 48 h; Retention Time: 8.805 min; Peak area: 5293329 volts. h: Fermentation time: 72 h; Retention Time: 8.813 min; Peak area: 4942502 volts. i: Fermentation time: 96 h; Retention Time: 8.663 min; Peak area: 5256189 volts. j: Fermentation time: 120 h; Retention Time: 8.565 min; Peak area: 4849635 volts.


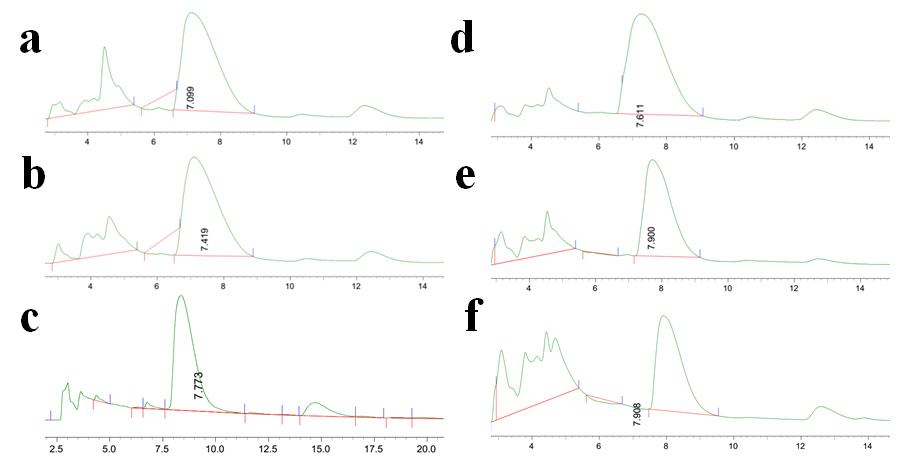


**Fig. S14 HPLC of effects of different precursor substances (urea and acetic acid) and its concentration on Xcn1 production.**

a: Urea: 2 mmol/L; Retention Time: 7.099 min; Peak area: 16246743 volts. b: Urea: 3 mmol/L (repeat 3); Retention Time: 7.419 min; Peak area: 17677852 volts. c: Urea: 4 mmol/L; Retention Time: 7.773 min; Peak area: 11410385 volts. d: Acetic acid: 2 mmol/L; Retention Time: 7.611 min; Peak area: 16703045 volts. e: Acetic acid: 3 mmol/L; Retention Time: 7.900 min; Peak area: 4153387 volts. f: Acetic acid: 4 mmol/L; Retention Time: 7.908 min; Peak area: 3601780 volts.


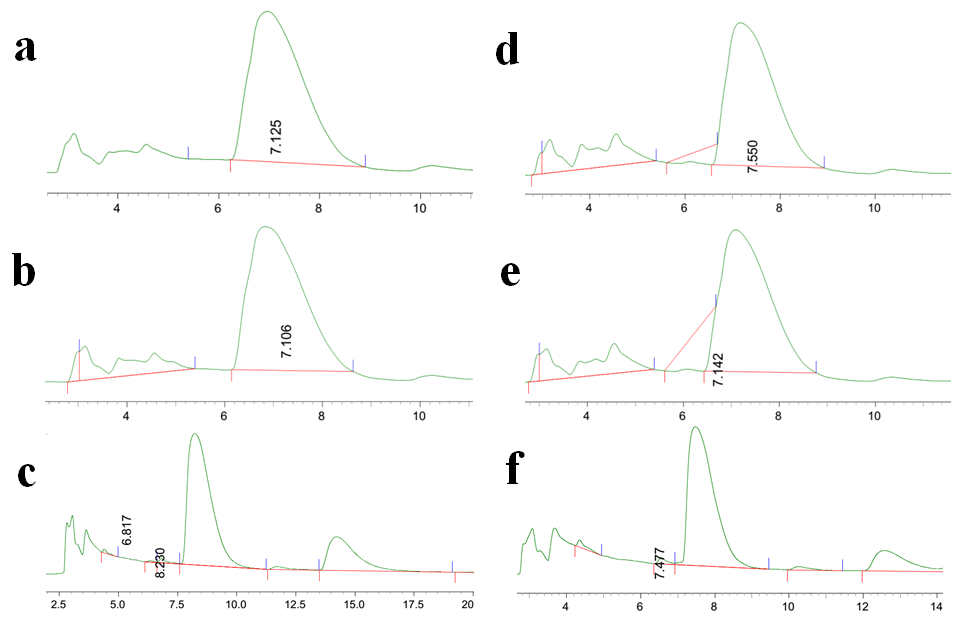


**Fig. S15 HPLC of effects of different precursor substances (arginine and leucine) and its concentration on Xcn1 production.**

a: Arginine: 2 mmol/L; Retention Time: 7.125 min; Peak area: 17475857 volts. b: Arginine: 3 mmol/L; Retention Time: 7.106 min; Peak area: 17680415 volts. c: Arginine: 4 mmol/L; Retention Time: 8.230 min; Peak area: 15504414 volts. d: Leucine: 2 mmol/L; Retention Time: 7.550 min; Peak area: 13939415 volts. e: Leucine: 3 mmol/L; Retention Time: 7.142 min; Peak area: 14591973 volts. f: Leucine: 4 mmol/L; Retention Time: 7.477 min; Peak area: 12794034 volts.


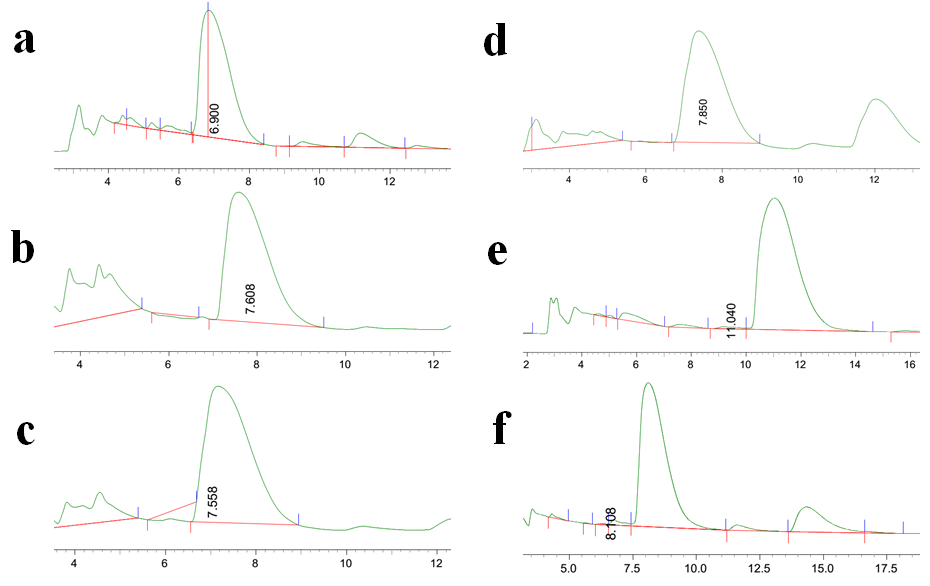


**Fig. S16 HPLC of effects of different adding time of arginine on Xcn1 production.**

a: Adding time of arginine: 0^th^ h; Retention Time: 6.900 min; Peak area: 18013437 volts. b: Adding time of arginine: 6^th^ h; Retention Time: 7.608 min; Peak area: 12427039 volts. c: Adding time of arginine: 12^th^ h; Retention Time: 7.558 min; Peak area: 23597608 volts. d: Adding time of arginine: 18^th^ h; Retention Time: 7.850 min; Peak area: 19817983 volts. e: Adding time of arginine: 24^th^ h; Retention Time: 11.040 min; Peak area: 15726665 volts. f: Adding time of arginine: 30^th^ h; Retention Time: 8.108 min; Peak area: 14565267 volts.

**Table S1** The data analysis of ^1^H NMR and ^13^C NMR spectra for Xcn1

| Carbon signals | | Proton signals | | |
| --- | --- | --- | --- | --- |
| Number | δ_C_ (ppm) | Number | δ_H_ (ppm) | Coupling constant (*J*, Hz) |
| C-1 | 169.10 | H-3 | 4.69-4.79 (1H) | m |
| C-3 | 80.34 | H-4 | 3.07-3.10 (2H) | m |
| C-4 | 28.10 | H-6 | 6.91 (1H) | d, *J* = 7.4 |
| C-5 | 138.80 | H-7 | 7.56 (1H) | t, *J* = 7.9 |
| C-6 | 118.18 | H-8 | 6.94 (1H) | d, *J* = 8.5 |
| C-7 | 136.00 | H-1’ | 0.89 (3H) | d, *J* = 6.6 |
| C-8 | 114.41 | H-2’ | 0.97 (3H) | d, *J* = 6.6 |
| C-9 | 159.00 | H-3’ | 1.50 (1H) | td, *J* = 9.6, 4.7 |
| C-10 | 107.11 | H-4’ A | 1.66-1.59 (1H) | m |
| C-1’ | 19.60 | H-4’ B | 1.73-1.66 (1H) | m |
| C-2’ | 21.39 | H-5’ | 4.27 (1H) | dt, *J* = 9.8, 4.8 |
| C-3’ | 23.15 | H-8’ | 4.33 (1H) | d, *J* = 6.0 |
| C-4’ | 37.44 | H-9’ | 4.17 (1H) | dd, *J* = 6.0 |
| C-5’ | 48.52 | H-10’ | 3.53 (1H) | td, *J* = 9.8, 6.6 |
| C-7’ | 172.50 | H-11’ A | 1.95-1.87 (1H), | m |
| C-8’ | 71.52 | H-11’ B | 1.85-1.74 (1H) | m |
| C-9’ | 69.45 | H-12’ | 1.85-1.74 (2H) | m |
| C-10’ | 52.49, | H-13’ | 3.27 (2H) | t, *J* = 6.6 |
| C-11’ | 28.10 |  |  |  |
| C-12’ | 23.41 |  |  |  |
| C-13’ | 39.54 |  |  |  |
| C-15’ | 155.67 |  |  |  |

Note: m: multiple peak; d: double peak; t: triplet peak; dd: double double peak; dt: double triplet peak; td: triplet double peak.
